# *C. elegans* under starvation produce proteinaceous material that supports collective aggregation into web-like structures

**DOI:** 10.17912/micropub.biology.001940

**Published:** 2025-12-16

**Authors:** May Li, Rex Kerr, Saul Kato

**Affiliations:** 1 Neurology, University of California, San Francisco, San Francisco, California, United States

## Abstract

We observed the aggregation of starved *C. elegans* into web-like patterns scaffolded by an unknown chemoattractive extracellular substance that is protein-rich, mucoid, water-insoluble, elastic, and likely to be both secreted and consumed by *C. elegans* . Under time-lapse imaging, we observed the formation of both the aggregation structures and the proteinaceous substance after populating an NGM plate with 100-200 worms and letting them starve over seven days. We preliminarily characterized the substance using Coomassie, WGA, and DAPI staining. We surmise that the substance may be composed of cuticles, remnants of dead worms, and worm yolk.

**Figure 1. C. elegans produce an extracellular proteinaceous material that forms complex spatial structures under starvation conditions:**
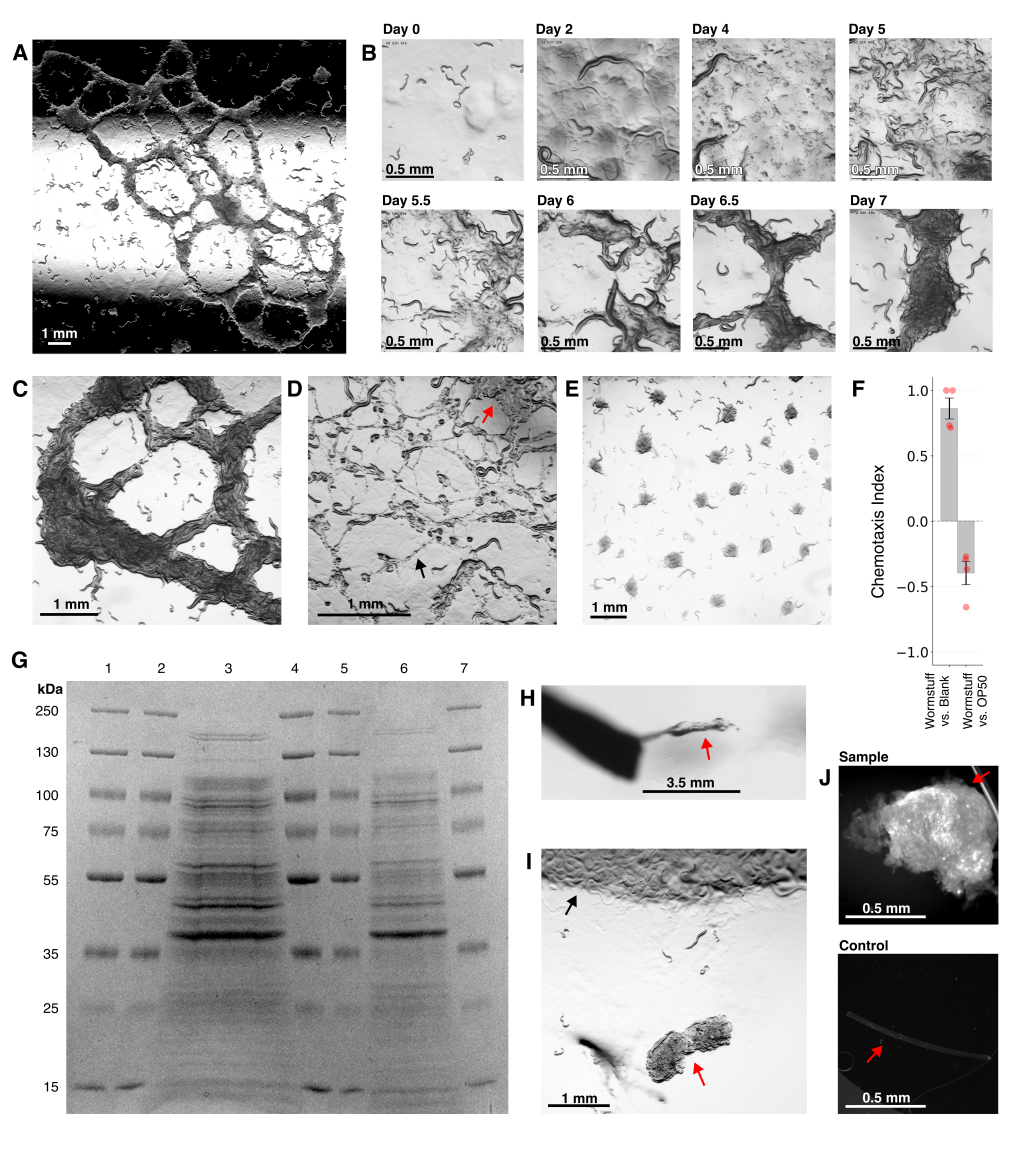
A) Mature worm web: worms aggregating around thread-like proteinaceous structures 7 days after introduction to an unseeded NGM plate. Illumination mode chosen to enhance visibility of 3D structure. B) Timelapse showing progression of worm web formation. 100-200 mixed-stage starved FOC126 worms were placed on an unseeded NGM plate. Bacterial growths are from residual bacteria on the animals. C) Illustration of thick rope-like worm web on day 7 after the introduction of worms. D) Intermediate stage of worm web formation on day 6. Note both thin thread-like structures (black arrow) and thicker mats of proteinaceous material (red arrow). E) Starvation-induced worm aggregation under typical lab culture conditions. 100-200 mixed-stage well-fed FOC126 worms were transferred to an NGM plate two days after seeding with 200 μL OP50 . Image taken 7 days after worm transfer. F) Chemotaxis to proteinaceous material versus blank control or OP50 , scored after 2h. Both starved FOC126 worms picked from worm webs and well-fed N2 worms were used, but results did not differ appreciably so are combined here. n=4 plates per condition; bar indicates mean and SEM. G) Coomassie gel electrophoresis of proteinaceous material. Lane 3, soluble fraction. Lane 6, insoluble fraction. Other lanes, molecular weight ladder. H) The proteinaceous material (red arrow) is cohesive and elastic, adhering to a platinum wire pick and stretching when lifted (see video in Extended Data). I) Retention of worms in proteinaceous material despite availability of food. Image taken 24h after placing a sample (red arrow) comprising proteinaceous material and worms adjacent to an OP50 bacterial lawn (black arrow). J) Top panel: high-magnification view of wormstuff (sample, red arrow) with WGA fluorescence labeling of glycoproteins and glycolipids on the cell surface. Bottom panel: high-magnification view of a human hair (control, red arrow) showing minimal non-specific WGA binding. Images were acquired using fluorescence stereo microscopy.

## Description

We observed the formation of interconnected web-like structures of aggregated mixed-age *C. elegans* approximately 7 days after seeding 100-200 starved worms on OP50 plates ( Fig. 1A ). The structures, which we term *worm webs* , bear some resemblance to previously reported transient spatial structures of worms. Transient structures have been induced with starved culture conditions combined with factors such as ethanol, humidity, population density, or condensation (Artyukhin et al., 2015, Sugi et al., 2019, Artyukhin et al., 2013, Chen et al., 2021), or result from collective feeding behavior&nbsp; (Demir et al., 2020, Ding et al., 2019). However, unlike other reported structures, worm webs maintained their geometry for days. Closer inspection of the worm webs revealed an extracellular proteinaceous biomaterial, which we term *wormstuff* , upon which the animals aggregated. We reliably reproduced worm webs using three different strains: N2 , FOC126, and FOC134, for a total of 60 replicates.

Time-lapse video and visual assessment of multiple plates revealed that the formation of worm webs occurs in a stereotypical sequence of visually distinctive phases ( Fig. 1B ; Extended Data). Initially, worms disperse throughout the plate in typical foraging behavior ( Fig. 1B, Day 0). New colonies of bacteria then grow, presumably as a result of residual bacteria deposited from the gut or cuticle from prior exposure to OP50 , providing sufficient food for animals to reach adulthood and lay eggs ( Fig. 1B, Day 2 and Day 4). As progeny begin to exhaust the food, thick mucoid deposits of material that differ in shape and color from existing bacteria begin to appear ( Fig. 1B, Day 5; Fig. 1D, red arrow). Worms aggregate upon these patches of material, which narrow into thin threads or coalesce into broader ropes, resulting in a web-like structure ( Fig. 1B, Day 5.5 and 6; Fig. 1D ). When bacteria are exhausted, the majority of the worm population aggregates upon this collective structure while displaying active pharyngeal pumping and webs often thicken and consolidate ( Fig. 1B, Day 6.5 and 7; Fig. 1C ). The structure then gradually disappears as the population continues to starve, suggesting that the worms can consume the material, albeit slowly relative to typical bacterial consumption rate.

This web formation is markedly different from starvation in typical culture conditions where worms are introduced to a seeded plate, starve, and aggregate into disk-shaped clusters that do not contain external material ( Fig. 1E ; Artyukhin et al, 2015). Worm webs differ from previously reported collective structures because they are stable for multiple days and appear to rely on an external substance (Chen et al., 2021, Sugi et al., 2019, Artyukhin et al, 2015). To assess whether wormstuff was bacterial or fungal in nature, we isolated the material on separate plates where it maintained its shape, neither growing nor shrinking. When not sterilized, bacterial colonies grew around it; heat sterilization (5 minutes in PBS buffer at 98°C) prevented bacterial growth. We then performed spinning disk confocal imaging of the sample with DAPI staining and observed no obvious cellular structures or nuclei. Thus, we tentatively conclude that wormstuff is not predominantly bacteria or fungi, and therefore is either excreted by worms or composed of worm remnants.&nbsp;

We conducted a preliminary investigation of the structural and biochemical properties of wormstuff. When it first appears in pools ( Fig. 1D, red arrow), the material is viscous, gel-like,&nbsp; and difficult to isolate with a pick. The initial thin thread-like strands ( Fig. 1D, black arrow) were also too sparse to harvest easily. However, on around Day 6, elastic masses of wormstuff visible to the naked eye could be lifted up using a worm pick while maintaining its integrity ( Fig. 1H ) and strands could be amassed. We found that wormstuff is not readily water-soluble and remains intact when submerged in distilled H _2_ O, which allows removal of worms by washing. However, heat treatment altered its structure: after 5 minutes at 98°C in buffer and reducing agent, the material separated into a soluble supernatant and insoluble dense pellet.

To test wormstuff for protein components, we first confirmed protein existence with a BCA assay, then ran an SDS-PAGE Coomassie gel to assess the distribution of protein sizes present ( Fig. 1G ). We observed a complex banding pattern that was less complex than that of whole-animal gels, but too complex to suggest or confirm individual protein constituents. We also stained, washed, and processed Day 8 wormstuff samples with wheat germ agglutinin (WGA) stain and found that the sample fluoresced brightly, indicating high glycoprotein/glycolipid content ( Fig. 1J, Sample, red arrow) compared to a negative control of a human hair ( Fig. 1J Control, red arrow).

One candidate component of wormstuff is worm yolk. Worm yolk is produced by post-reproductive adults which convert their intestines to yolk, then continuously vent the yolk to provide a nutritional resource for progeny (Kern et al., 2021), peaking at days 4-6 of adulthood. Another candidate component of wormstuff is molted cuticles or cuticular proteins; cuticles consist of glycoproteins, glycolipids, cross-linked collagens, and insoluble proteins (Page et al., 2007) and are shed repeatedly during development. A third candidate is worm corpses. During acute starvation, many *C. elegans* strains are known to bag: larvae hatch inside the mother, consume its internal contents, and leave behind an empty cuticle (Chen et al., 2004). Although we observed bagged worms on worm webs from Day 7 onwards, we observed initial deposits of wormstuff at around Day 4 before the development of worm bagging; thus, wormstuff is not likely to be composed exclusively of worm corpses.

Perhaps the most striking characteristic of the worm webs is that the worms aggregate on and within wormstuff ( Fig. 1A, Days 5+; Fig. 1C ; and Extended Data). When we transferred wormstuff and worms from a starved plate onto a seeded plate, many worms remained in the wormstuff rather than migrating to the OP50 lawn ( Fig. 1I ). In chemotaxis assays, worms strongly preferred wormstuff to an empty target area, but moderately preferred OP50 over wormstuff ( Fig. 1F ), indicating that attraction to wormstuff likely plays a role in the aggregation behavior. This attraction may not be to the proteinaceous material itself even though the animals appear to gradually consume the material; for instance, worm yolk is not chemoattractive even though it is consumed by larvae (Kern et al., 2021). It is also possible that the material becomes saturated with pheromones from worms, bacterial odors, or other small molecules. After treatment in PBS buffer for 5 minutes at 98°C, wormstuff no longer appeared to attract worms, suggesting either a change in composition or that the treatment removed chemoattractive cues.

We observed collective behavior of aggregation upon a chemoattractive proteinaceous substrate that likely originates from worms, creating three-dimensional spatial structures over the course of 7 days. Worm webs are a geometrical intermediate between dispersal and point-like aggregation. We hypothesize that the production of wormstuff-supported worm webs represents an adaptive strategy to extend the spatial coverage and prolong the lifetime of a pre-dauer (i.e. actively feeding) colony.

## Methods

**Worm Culture Conditions**

*C. elegans* strains were maintained at 20°C on nematode growth medium (NGM) plates seeded with *Escherichia coli* OP50 according to standard protocols (Stiernagle et al., 2006). To generate worm webs, 100-200 mixed-age unwashed worms were picked from either a prior starved or unstarved plate, introduced to unseeded NGM plates, and left for 7 days in 20°C. On the seventh day, wormstuff was gathered for assays. We found that while picking worms from plates that already contained worm webs would guarantee formation of a new worm web, doing so is not required to induce worm web formation. We presume that the emergence of bacterial colonies observed in the process of worm web formation, as evident in the timelapse video (Extended Data), was the result of residual bacteria carried over on or in the worms.

**Chemotaxis Assays**

Chemotactic attraction to wormstuff was quantified using a two-choice assay. Mixed-stage worms (100-200 worms per plate), either well-fed N2 or starved FOC126 from web-forming plates, were placed in a central loading zone on unseeded NGM plates. On opposite sides of the plate, two target areas were selected.&nbsp; In one, we placed isolated wormstuff (approximately 0.1 μL) 3 hours before the start of the assay.&nbsp; The other we either left blank or seeded with 1 μL of OP50 24 hours before the start of the assay. After 2 hours at room temperature, worms that migrated to each target area were counted. The chemotaxis index (CI) was calculated as:

CI = (# worms at wormstuff target − # worms at other target) / total # of worms

Four replicates were performed for each condition, two with well-fed N2 and two with starved FOC126; these did not differ dramatically from each other and thus were grouped. CI values range from -1 (complete aversion) to +1 (complete attraction), with 0 indicating no preference. Statistical significance was assessed using one-sample t-tests comparing CI to zero (no preference).

**Imaging and Microscopy**

Images of worm webs were captured on a Leica M165 FC stereo microscope equipped with a Leica K5 camera. Timelapse recordings were captured on a Leica M205 FCA stereo microscope with an Orca Flash 4.0 C13440 camera. High-resolution fluorescence imaging was performed using a Yokogawa W1-SoRa spinning disk confocal microscope. Contrast adjustments, when needed for clarity, were performed using ImageJ/FIJI version 2.16. Timelapse videos were extracted from Leica .lif files using a custom utility written in Scala 3.7 and utilizing BioFormats 8.3 image readers (available at https://github.com/focolab/lif-timelapse-to-mp4/releases/tag/v0.1.0 ). Movies were encoded using ImageJ/FIJI or the Bytedeco JavaCV FFmpeg API version 1.5.

**Protein Extraction and Gel Electrophoresis**

The wormstuff material was mechanically isolated from plates using platinum wire picks and washed three times with distilled H _2_ O to remove adherent bacteria and worms. The isolated material was transferred to microcentrifuge tubes containing RIPA lysis buffer (50 mM Tris-HCl pH 8.0, 150 mM NaCl, 1% NP-40, 0.5% sodium deoxycholate, 0.1% SDS) supplemented with protease inhibitor cocktail.

Samples were sonicated using a probe sonicator at amplitude 1 for three 5-second bursts with 30-second intervals. Following sonication, lysates were centrifuged at 17,000 × g for 10 minutes at 4°C to separate soluble and insoluble fractions. The supernatant (soluble fraction) was carefully removed and transferred to a new tube.

The remaining pellet (insoluble fraction) was subjected to additional extraction by resuspending in 2% SDS buffer and incubating at 55°C for 10 minutes with periodic vortexing. Following this treatment, samples were centrifuged again at 17,000 × g for 10 minutes at 4°C, and the supernatant representing the SDS-solubilized portion of the insoluble fraction was collected.&nbsp; Some material remained in the pellet. Both soluble and insoluble fractions were quantified using the Pierce BCA Protein Assay Kit.

Samples were prepared for electrophoresis by adding NuPAGE LDS Sample Buffer (4×) and NuPAGE Sample Reducing Agent (10×) to achieve final concentrations of 1× each. Samples were denatured by heating at 98°C for 5 minutes, which both unfolds proteins and reduces disulfide bonds to linearize the polypeptide chains.&nbsp;

Proteins were separated by SDS-PAGE using precast NuPAGE 4-20% Bis-Tris protein gels with 10 wells. Electrophoresis was performed in NuPAGE MES SDS Running Buffer at 200 V for approximately 45 minutes until the dye front reached the bottom of the gel. Protein molecular weight markers (PageRuler Plus, Thermo Fisher Scientific) were loaded in lanes 1, 2, 4, 5, and 7 to enable molecular weight determination.

Following electrophoresis, gels were stained with Coomassie Brilliant Blue R-250 using a standard staining protocol. Briefly, gels were fixed in 40% methanol and 10% acetic acid for 30 minutes, stained in Coomassie Brilliant Blue R-250 solution for 1 hour, and destained in 40% methanol and 10% acetic acid until protein bands were clearly visible against a clear background.

**Wheat Germ Agglutinin (WGA) Fluorescence Staining** To detect glycoproteins and glycolipids in the wormstuff, samples were stained with fluorescently conjugated wheat germ agglutinin (WGA), which binds to N-acetylglucosamine and sialic acid residues. Isolated wormstuff was fixed with 4% paraformaldehyde in phosphate-buffered saline (PBS) for 30 minutes at room temperature.

Fixed samples were permeabilized with 0.2% Triton X-100 in PBS for 20 minutes to allow antibody penetration, followed by one PBS wash. Samples were then incubated with WGA conjugated to PhenoVue Fluor 488 fluorophore (20 μg/mL in PBS) for 30 minutes at room temperature in the dark. Following two final PBS washes to remove unbound WGA, samples were mounted on glass slides using DAPI Fluoromount-G mounting medium with spacers to prevent compression of three-dimensional structures. As a negative control, human hair samples were processed identically to test for non-specific binding of WGA.

Images of stained material were captured on the spinning disk confocal and assessed visually. Staining was bright but amorphous so the images were not analyzed further.

**DAPI Staining**

To assess whether the proteinaceous material contained living cells or contaminating organisms, samples were stained with 4',6-diamidino-2-phenylindole (DAPI). Isolated wormstuff was fixed with 4% paraformaldehyde in PBS for 30 minutes at room temperature.

Samples were permeabilized with 0.2% Triton X-100 in PBS for 20 minutes to allow DAPI penetration, followed by two PBS washes.&nbsp;

Samples were mounted directly using DAPI Fluoromount-G mounting medium without spacers and imaged on a spinning disk confocal. Staining was dim, even at maximum laser power, and amorphous, so the images were not analyzed further.

## Reagents

**Table:** 

Strain	Genotype	Corresponding Injection Plasmid	Available from
N2	*Caenorhabditis elegans*	N/A	CGC
FOC126	OH16230 (NeuroPAL + GCaMP6s); Ex[pFC078]	pFC078 osm-10p::WOrMsChRmine::wrmScarlet @ 5ng/uL	focolab.org
FOC134	OH16230 (NeuroPAL + GCaMP6s); Ex[pFC082+ unc-122 ::RFP]	pFC082odr-10p::WOrMsChRmine::wrmScarlet@ 2.5ng/uL + unc-122 RFP@ 50ng/uL	focolab.org

## Data Availability

Description: Timelapse video of worm web formation, 0-7 days, 6000x speed (10 minutes/frame). Resource Type: Audiovisual. DOI: https://doi.org/10.22002/40m20-r7410 Description: Manipulation of wormstuff with platinum wire pick to illustrate mechanical properties. Resource Type: Audiovisual. DOI: https://doi.org/10.22002/08jc4-cyw35
